# Relationship between MLH1, MSH2, MSH6, and PMS2 protein expression status and clinicopathological characteristics in colorectal cancer tissues

**DOI:** 10.3389/fmed.2026.1840244

**Published:** 2026-06-12

**Authors:** Wansheng Hu, Xiaowei Hu, Enyun Xie, Yanyan Wang

**Affiliations:** Department of Pathology, Zongyang County People’s Hospital, Tongling, Anhui, China

**Keywords:** clinicopathological characteristics, colorectal cancer, immunohistochemistry, mismatch repair proteins, MLH1, MSH2, MSH6, PMS2

## Abstract

**Background:**

Deficient mismatch repair (dMMR) is a key molecular subtype of colorectal cancer (CRC) with significant prognostic and therapeutic implications, particularly regarding response to immunotherapy. The prevalence and clinicopathological features of dMMR CRC can vary by population. This research aimed to assess the prevalence of MMR protein deficiency and its relation to clinicopathological characteristics in Chinese CRC patients.

**Methods:**

Eighty patients with primary colorectal adenocarcinoma who underwent radical surgical resection were enrolled. Immunohistochemistry (IHC) was used to detect the expression of the four MMR proteins (MLH1, MSH2, MSH6, and PMS2). Patients were classified as dMMR or pMMR. Associations between MMR status and various clinicopathological features, including tumor location, differentiation, histology, and lymph node metastasis, were analyzed using Fisher’s exact or chi-square tests, Spearman correlation, and simplified multivariable logistic regression with forward stepwise selection.

**Results:**

Of the 80 patients, 18 (22.5%) were identified as dMMR. The most common deficiency pattern was isolated PMS2 loss (50.0% of dMMR cases), followed by combined loss of MLH1/PMS2 (33.3%). Compared to the pMMR group, dMMR CRCs were significantly more likely to be located in the right colon (61.1% vs. 22.6%, *P* < 0.001), be poorly differentiated (50.0% vs. 19.4%, *P* = 0.009), exhibit mucinous adenocarcinoma (27.8% vs. 6.5%, *P* = 0.014), and be larger in size (≥ 5 cm: 55.6% vs. 30.6%, *P* = 0.048). Conversely, dMMR status was linked to a suggestively significantly lower rate of lymph node metastasis (22.2% vs. 46.8%, *P* = 0.052). Forward stepwise multivariable analysis confirmed right colon location (OR = 4.12), poor differentiation (OR = 3.45), and mucinous histology (OR = 3.01) as independent predictors of dMMR. Notably, isolated MSH6 loss was suggestively associated with younger age and poor differentiation, but these findings are preliminary due to the small number of cases.

**Conclusion:**

The prevalence of dMMR in this cohort was 22.5%. dMMR CRCs display a distinct clinicopathological profile characterized by right-sided location, poor differentiation, mucinous histology, larger tumor size, along with a lower likelihood of lymph node metastasis. These findings support the value of routine IHC-based MMR testing in CRC patients. However, confirmatory molecular testing (e.g., MSI analysis, BRAF V600E mutation, MLH1 promoter methylation, or germline sequencing) is necessary to differentiate sporadic from Lynch syndrome-associated dMMR cases and to fully guide Lynch syndrome screening and immunotherapy decisions.

## Introduction

Colorectal cancer (CRC) is one of the most common malignant tumors worldwide, ranking third in incidence and second in mortality among all cancers according to the 2020 Global Cancer Statistics report, with around 1.9 million new cases as well as over 900,000 deaths annually (1). The occurrence and development of CRC involve complex molecular mechanisms, including chromosomal instability, microsatellite instability (MSI), and CpG island methylator phenotype (2). Among these, MSI caused by DNA mismatch repair (MMR) deficiency plays a crucial role in approximately 12–15% of CRC cases, encompassing both Lynch syndrome (about 3%) and sporadic MSI CRC (about 12%) (3).

The MMR system maintains genomic stability by recognizing and repairing errors that occur during DNA replication, containing base-base mismatches and insertion-deletion loops (4). The key proteins involved in this process include MLH1, MSH2, MSH6, and PMS2, which form functional heterodimers: MLH1 dimerizes with PMS2, while MSH2 dimerizes with MSH6 (5). When any of these MMR genes undergo germline mutation, somatic mutation, or epigenetic inactivation (such as MLH1 promoter hypermethylation), the corresponding protein expression is lost, leading to deficient mismatch repair (dMMR) and subsequent microsatellite instability-high (MSI-H) phenotype (6).

Immunohistochemical (IHC) detection of MMR protein expression has become routine due to its simplicity, reliability, and cost-effectiveness compared to genetic analysis (7). Complete loss of nuclear staining in tumor cells for any of the four MMR proteins indicates dMMR, while preserved expression indicates proficient mismatch repair (pMMR) (8). This testing not only serves as a screening tool for Lynch syndrome but also provides crucial predictive information for immunotherapy response, as dMMR/MSI-H tumors have demonstrated remarkable sensitivity to immune checkpoint inhibitors regardless of tumor origin (9). Recent studies have shown that immunotherapy drugs targeting PD-1 can achieve remarkable responses in dMMR CRC patients, with some even achieving complete pathological responses without requiring prolonged treatment (10).

Previous studies have reported that dMMR CRCs exhibit distinct clinicopathological characteristics compared to pMMR CRCs, including predilection for the proximal colon, poor differentiation, mucinous histology, prominent tumor-infiltrating lymphocytes, and more favorable prognosis (11). However, the prevalence of dMMR and its link with clinicopathological features may vary across diverse populations and geographic regions. A large-scale study from Changsha reported a dMMR rate of 16.9% in 855 CRC cases (12), while a study from Beijing, China reported dMMR rates of 27.19% in 228 CRC patients (13). These variations highlight the significance of conducting region-specific researches to understand the characteristics of dMMR CRC in different populations.

This study aims to comprehensively assess the expression status of MLH1, MSH2, MSH6, and PMS2 proteins and their relationship with various clinicopathological characteristics, providing a theoretical basis and clinical reference for precision medicine approaches in CRC management.

## Materials and methods

### General information

Eighty patients with pathologically confirmed CRC who experienced surgical resection in our hospital from October 2023 to December 2025 were chosen as research participants. Inclusion criteria: (1) pathologically confirmed primary colorectal adenocarcinoma; (2) underwent radical surgical resection; (3) complete clinical and pathological data available; (4) no preoperative radiotherapy or chemotherapy. Exclusion criteria: (1) recurrent colorectal cancer; (2) multiple primary colorectal cancers; (3) combined with other malignant tumors; (4) history of neoadjuvant therapy; (5) incomplete clinical or pathological data; (6) inadequate tissue samples for IHC analysis. This study was approved by the hospital ethics committee, and all patients provided informed consent.

### Immunohistochemical detection of MMR protein expression

Formalin-fixed, paraffin-embedded (FFPE) tissue blocks preserved in formalin and embedded in paraffin (FFPE), derived from surgical resection specimens, were gathered. Sections with a thickness of four micrometers were sliced and affixed to positively charged glass slides. Immunohistochemical staining was performed using the EnVision two-step technique on an automated immunostainer (Dako Autostainer Link 48, Agilent, Santa Clara, CA, United States). Primary antibodies targeting MLH1 (clone ES05), MSH2 (clone FE11), MSH6 (clone EP49), and PMS2 (clone EP51) were utilized at the following dilutions: MLH1 (1/5,000), MSH2 (1/2,000), MSH6 (1/500), and PMS2 (1/2,000) (all from Thermo Fisher, United States).

Briefly, sections underwent deparaffinization, rehydration, heat-induced epitope retrieval (EnVision FLEX Target Retrieval Solution, high pH, 97°C for 20 min), and peroxidase blocking (3% hydrogen peroxide for 10 min). Sections were then incubated with primary antibodies (room temperature, 30 min), followed by HRP-conjugated polymer (EnVision FLEX/HRP, Dako) for 30 min. Diaminobenzidine was used as chromogen, and hematoxylin for counterstaining.

Positive and negative controls: For each staining run, normal colonic mucosa (which expresses all four MMR proteins) served as an external positive control. Additionally, internal positive controls consisting of non-neoplastic colonic epithelial cells, lymphocytes, and stromal cells were assessed in each tissue section. Negative controls were prepared by substituting the primary antibody with phosphate-buffered saline (PBS) or an irrelevant isotype-matched antibody.

Two seasoned pathologists independently assessed the immunohistochemical staining results without access to the clinical data. Nuclear staining of any intensity (weak, moderate, or strong) in tumor cells, whether focal (≥ 1% of tumor nuclei) or patchy, was considered indicative of “no loss of expression” (preserved expression), in accordance with the College of American Pathologists (CAP) guidelines. Only a complete absence of nuclear staining in all tumor cells, with intact nuclear staining in internal positive controls, was deemed “loss of expression” (deficient expression). Because any nuclear staining (regardless of intensity or percentage of positive tumor cells) was classified as preserved expression, and only complete absence of nuclear staining in all tumor cells was classified as loss, no equivocal or indeterminate cases were encountered. This binary classification system eliminated the need for an intermediate category. Cases with discrepant evaluations were re-examined collaboratively to achieve a consensus. Interobserver agreement between the two pathologists was assessed using percent agreement and Cohen’s kappa (κ) coefficient, calculated on a case-by-case basis for each MMR protein.

### Clinicopathological data collection

The following clinicopathological characteristics were collected from medical records, containing demographic characteristics (gender, age), tumor characteristics (tumor location, tumor size, histological type, differentiation grade), pathological staging (depth of invasion, lymph node metastasis, TNM stage), other pathological features (vascular invasion, neural invasion) as well as laboratory parameters [preoperative carcinoembryonic antigen (CEA) levels].

### Statistical analysis

SPSS 26.0 statistical software was implemented for data analysis. Measurement data were exhibited as mean ± standard deviation (x̄ ± s), and comparisons between groups were made using independent samples *t*-test. Count data were exhibited as number of cases and percentages (%). For comparisons between the dMMR and pMMR groups, the chi-square (χ^2^) test was used when all expected cell counts were ≥ 5; otherwise, Fisher’s exact test was applied. Spearman rank correlation analysis was performed to evaluate the correlation between MMR protein expression status (dMMR vs. pMMR) and various clinicopathological characteristics.

For multivariable analysis, variables with *P* < 0.10 in univariate analysis (tumor location, tumor size, histological type, differentiation grade, and lymph node metastasis) were considered for entry. To avoid overfitting given the limited number of dMMR cases (*n* = 18), we performed forward stepwise logistic regression (entry criterion *P* < 0.05, removal criterion *P* > 0.10). The final model retained only variables that remained independently significant. The events-per-variable (EPV) ratio was calculated to assess model stability. Given the exploratory nature of this study, no adjustment for multiple comparisons was applied; thus, findings with *P*-values near 0.05 should be interpreted cautiously and require validation in larger independent cohorts. *P* < 0.05 was considered statistically significant for univariate analyses, while in the multivariable model, a more conservative threshold (*P* < 0.05) was maintained with attention to EPV.

## Results

### General characteristics of the study population and MMR protein expression profiles

A total of 80 CRC patients were included in this study, containing 50 males (62.5%) and 30 females (37.5%), with a mean age of 59.1 ± 10.8 years (range: 35–82 years). Tumors were located in the right colon in 21 patients (26.3%), the left colon in 21 patients (26.3%), and the rectum in 38 patients (47.4%). Most tumors were non-mucinous (71/80, 88.8%), including 70 cases of adenocarcinoma and 1 case of other histology; mucinous adenocarcinoma was identified in 9 patients (11.3%). Regarding differentiation grade, 15 tumors (18.8%) were well-differentiated, 44 (55.0%) were moderately differentiated, and 21 (26.2%) were poorly differentiated.

**FIGURE 1:**
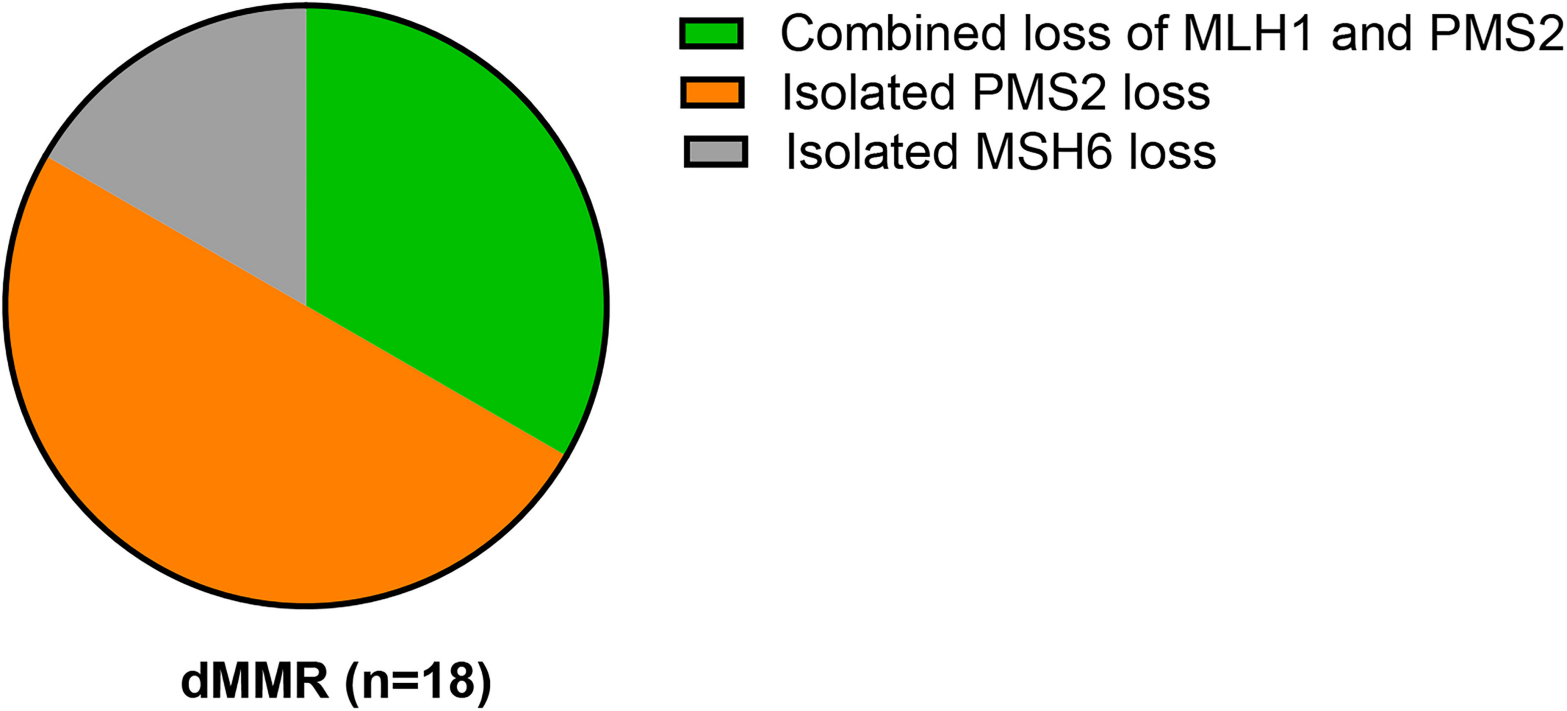
Distribution of MMR protein deficiency patterns in dMMR CRC cases.

Of the 80 patients, 18 (22.5%) were classified as having dMMR, while the remaining 62 (77.5%) were classified as having pMMR. Among the 18 dMMR cases, the most common pattern was isolated loss of PMS2 expression, observed in 9 patients (50.0% of dMMR cases). This was followed by combined loss of MLH1 and PMS2 in 6 patients (33.3%), and isolated loss of MSH6 in 3 patients (16.7%). No cases of isolated MSH2 loss or combined MSH2/MSH6 loss were observed in this cohort (Figure 1). No cases showed equivocal IHC staining patterns (e.g., weak/focal staining that could not be definitively classified as preserved or lost); all 80 cases were unequivocally assigned to either the dMMR or pMMR group based on the prespecified CAP criteria.

**FIGURE 2:**
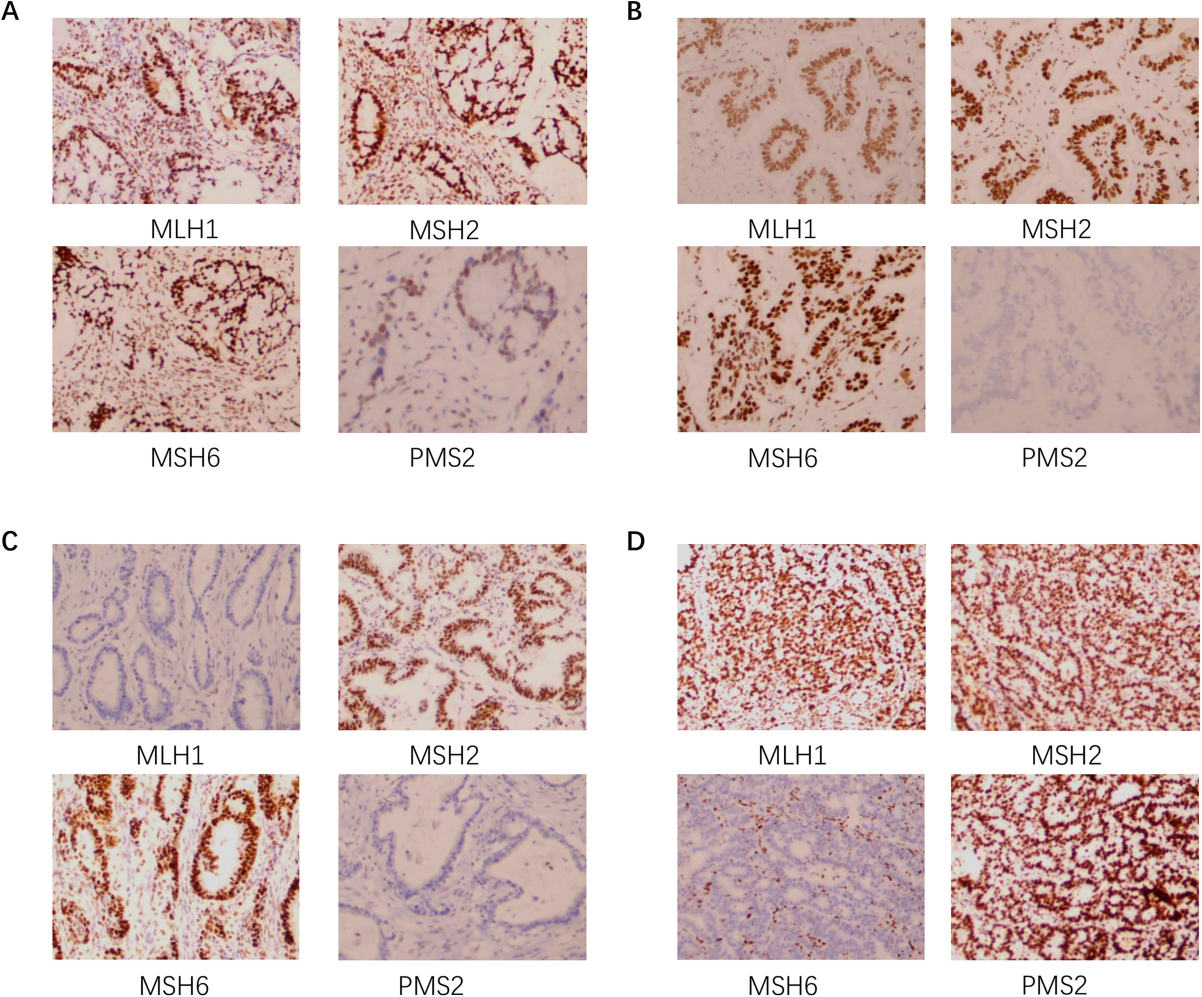
Representative immunohistochemical staining for MMR proteins in colorectal cancer tissues (original magnification × 400). **(A)** Preserved expression of MLH1, MSH2, MSH6, and PMS2 in a pMMR CRC. **(B)** Isolated loss of PMS2 expression with intact MLH1, MSH2, and MSH6. **(C)** Combined loss of MLH1 and PMS2 with intact MSH2 and MSH6. **(D)** Isolated loss of MSH6 with intact MLH1, MSH2, and PMS2. Brown nuclear staining indicates positive expression; absence of brown signal in tumor cells (with positive internal controls) indicates loss.

Representative immunohistochemical images of preserved MMR expression, isolated PMS2 loss, combined MLH1/PMS2 loss, and isolated MSH6 loss are shown in Figure 2.

### Interobserver agreement for MMR IHC interpretation

The two pathologists showed excellent agreement in interpreting MMR protein expression status. The overall percent agreement was 96.3% (77/80 cases) for MLH1, 98.8% (79/80) for MSH2, 95.0% (76/80) for MSH6, and 97.5% (78/80) for PMS2. Cohen’s kappa coefficients were 0.92 (95% CI: 0.84–1.00) for MLH1, 0.96 (95% CI: 0.89–1.00) for MSH2, 0.89 (95% CI: 0.79–0.99) for MSH6, and 0.94 (95% CI: 0.86–1.00) for PMS2, indicating almost perfect agreement for all four markers. Discrepant cases (*n* = 4 total) were resolved by joint review on a multi-head microscope, with consensus achieved in all instances.

### Comparison of clinicopathological characteristics between both groups

A comprehensive comparison of clinicopathological features between the dMMR (*n* = 18) and pMMR (*n* = 62) groups is presented in Table 1.

**TABLE 1:** Comparison of clinicopathological characteristics between dMMR and pMMR groups [n (%)]

Clinicopathological characteristic	dMMR group (*n* = 18)	pMMR group (*n* = 62)	Test	*P-*value
**Gender**			χ^2^	0.489
Male	10 (55.6)	40 (64.5)		
Female	8 (44.4)	22 (35.5)		
**Age (years)**			χ^2^	0.408
< 50	4 (22.2)	11 (17.7)		
50–59	5 (27.8)	19 (30.6)		
60–69	5 (27.8)	21 (33.9)		
≥ 70	4 (22.2)	11 (17.7)		
Tumor location			Fisher’s exact	**< 0.001**
Right colon	11 (61.1)	10 (16.1)		
Left colon	4 (22.2)	17 (27.4)		
Rectum	3 (16.7)	35 (56.5)		
Tumor size			χ^2^	**0.048**
< 5 cm	8 (44.4)	43 (69.4)		
≥ 5 cm	10 (55.6)	19 (30.6)		
Histological type			Fisher’s exact	**0.014**
Mucinous adenocarcinoma	5 (27.8)	4 (6.5)		
Non-mucinous	13 (72.2)	58 (93.5)		
Differentiation grade			χ^2^	**0.009**
Well-differentiated	2 (11.1)	13 (21.0)		
Moderately differentiated	7 (38.9)	37 (59.7)		
Poorly differentiated	9 (50.0)	12 (19.4)		
Depth of invasion (T stage)			χ^2^	0.499
T1-T2	5 (27.8)	21 (33.9)		
T3-T4	13 (72.2)	41 (66.1)		
Lymph node metastasis			Fisher’s exact	**0.052**
Negative (N0)	14 (77.8)	33 (53.2)		
Positive (N1-N2)	4 (22.2)	29 (46.8)		
TNM stage			χ^2^	0.128
Stage I	3 (16.7)	11 (17.7)		
Stage II	11 (61.1)	24 (38.7)		
Stage III	3 (16.7)	24 (38.7)		
Stage IV	1 (5.6)	3 (4.8)		
Vascular invasion	3 (16.7)	15 (24.2)	χ^2^	0.501
Neural invasion	2 (11.1)	14 (22.6)	χ^2^	0.285
CEA level			χ^2^	0.583
< 5 ng/mL	12 (66.7)	38 (61.3)		
≥ 5 ng/mL	6 (33.3)	24 (38.7)		

Bold values indicate *P* < 0.05.

Significant associations were found between dMMR status and several tumor characteristics. dMMR CRCs were predominantly located in the right colon (61.1% vs. 22.6%, *P* < 0.001, Fisher’s exact test) and were more likely to be poorly differentiated (50.0% vs. 19.4%, *P* = 0.009, χ^2^) and of mucinous histology (27.8% vs. 6.5%, *P* = 0.014, Fisher’s exact test). Furthermore, dMMR CRCs were significantly larger (≥ 5 cm: 55.6% vs. 30.6%, *P* = 0.048, χ^2^) and exhibited a suggestively lower rate of lymph node metastasis (22.2% vs. 46.8%, *P* = 0.052, Fisher’s exact test) relative to pMMR CRCs.

No differences were discovered between both groups regarding gender, age, depth of invasion (T stage), TNM stage (though a trend was noted), vascular invasion, neural invasion, or preoperative CEA levels (all *P* > 0.05).

### Correlation between dMMR status and clinicopathological characteristics

As shown in Table 2, dMMR status showed a significant positive correlation with right colon location (*r* = 0.335, *P* < 0.001), poor differentiation (*r* = 0.291, *P* = 0.009), mucinous histology (*r* = 0.278, *P* = 0.012), and larger tumor size (*r* = 0.221, *P* = 0.048). Conversely, a significant negative correlation was found between dMMR status and lymph node metastasis (*r* = -0.220, *P* = 0.049). No significant correlations were identified with other characteristics such as gender, age, or CEA levels (*P* > 0.05).

**TABLE 2:** Spearman correlation analysis between dMMR status and clinicopathological characteristics.

Clinicopathological characteristic	Correlation coefficient (r)	*P*-value
Gender (male vs. female)	-0.044	0.693
Age (≥ 60 vs. < 60 years)	-0.081	0.408
Tumor location (right colon vs. others)	0.335	< 0.001
Tumor size (≥ 5 cm vs. < 5 cm)	0.221	0.048
Histological type (mucinous vs. others)	0.278	0.012
Differentiation grade (poor vs. others)	0.291	0.009
T stage (T3-T4 vs. T1-T2)	0.076	0.499
Lymph node metastasis (positive vs. negative)	-0.220	0.049
Vascular invasion (present vs. absent)	-0.075	0.501
Neural invasion (present vs. absent)	-0.120	0.285
CEA level (≥ 5 vs. < 5 ng/mL)	-0.061	0.583

### Association of individual MMR protein loss with clinicopathological features

Analysis of specific MMR protein loss patterns revealed distinct associations with clinicopathological features (Table 3). MLH1 loss (observed in 6 cases, all with concurrent PMS2 loss) was significantly associated with right colon location, poor differentiation, and mucinous histology. PMS2 loss (observed in 15 cases, including 6 with MLH1 loss and 9 isolated) was significantly associated with right colon location, poor differentiation, and larger tumor size. MSH6 loss (observed in 3 cases, all isolated) was suggestively associated with younger age and poor differentiation, but these findings are preliminary due to the small number of cases.

**TABLE 3:** Association of individual MMR protein loss with key clinicopathological characteristics.

Protein loss (n)	Associated clinicopathological features	*P*-value
MLH1 loss (with PMS2) (*n* = 6)	Right colon location (83.3%)	0.002
	Poor differentiation (66.7%)	0.007
	Mucinous histology (33.3%)	0.021
MSH6 loss (isolated) (*n* = 3)	Younger age (< 50 years: 66.7%)	0.028
	Poor differentiation (100.0%)	0.015
PMS2 loss (*n* = 15)	Right colon location (66.7%)	< 0.001
	Poor differentiation (53.3%)	0.008
	Larger tumor size (≥ 5 cm: 60.0%)	0.032

### Multivariable logistic regression analysis for dMMR status

**FIGURE 3:**
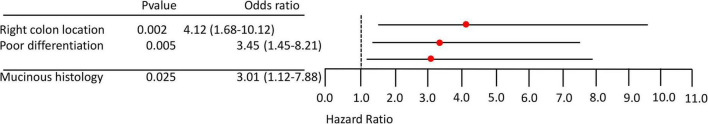
Logistic regression coefficient plot showing the independent predictors of dMMR status from forward stepwise multivariable analysis. Odds ratios (ORs) with 95% confidence intervals are displayed for right colon location, poor differentiation, and mucinous histology.

To identify independent predictors of dMMR status, forward stepwise logistic regression was implemented, incorporating variables with *P* < 0.10 in univariate analyses (tumor location, tumor size, histological type, differentiation grade, and lymph node metastasis). Given the limited number of dMMR events (*n* = 18), the stepwise procedure retained only three significant predictors, resulting in an events-per-variable ratio of 6:1. As detailed in Table 4, right colon location (OR = 4.12, 95% CI: 1.68–10.12, *P* = 0.002), poor differentiation (OR = 3.45, 95% CI: 1.45–8.21, *P* = 0.005), and mucinous histology (OR = 3.01, 95% CI: 1.15–7.88, *P* = 0.025) emerged as independent and significant predictors of dMMR status. Tumor size ≥ 5 cm and lymph node metastasis did not enter the final model (*P* > 0.10 after stepwise selection). A logistic regression coefficient plot visually representing these results is provided in Figure 3.

**TABLE 4:** Multivariable logistic regression analysis of factors associated with dMMR status.

Variable	B	SE	Wald χ ^2^	OR	95% CI	*P*-value
Right colon location	1.416	0.461	9.431	4.12	1.68–10.12	0.002
Poor differentiation	1.238	0.443	7.813	3.45	1.45–8.21	0.005
Mucinous histology	1.102	0.491	5.037	3.01	1.12–7.88	0.025

## Discussion

This research assessed the prevalence and clinicopathological significance of MMR protein deficiency in a cohort of 80 Chinese patients with CRC. Our findings reveal a dMMR prevalence of 22.5%, which is within the higher range of previously reported rates (12–15% globally) (14, 15). This relatively high prevalence underscores the importance of routine MMR testing in our population.

While IHC is a well-validated surrogate for MSI status, it is important to acknowledge that IHC and MSI testing (PCR or NGS) are not perfectly concordant. Reported discordance rates range from 1 to 5% of CRC cases, typically due to rare scenarios such as retained expression of a mutated but non-functional MMR protein (IHC false positive for pMMR) or loss of expression without microsatellite instability (e.g., due to truncating mutations in the 5’ region of MSH6 or certain MLH1 missense mutations). Conversely, MSI-high tumors with intact MMR protein expression on IHC have also been reported, often associated with MSH6 mutations or rare MSH2 missense variants. Our study did not perform confirmatory MSI PCR or NGS testing, which may have implications for the small subset of cases where IHC and MSI status would diverge. Consequently, for clinical decision-making—particularly regarding immunotherapy eligibility—some guidelines recommend reflex MSI testing when IHC results are equivocal or when there is strong clinical suspicion despite intact IHC staining. However, for routine Lynch syndrome screening and initial risk stratification, IHC remains an excellent first-line tool with high sensitivity and specificity. Future studies incorporating MSI analysis would help clarify the true dMMR prevalence in our population and resolve any potential discordant cases.

The most common pattern of MMR deficiency in our cohort was isolated loss of PMS2 (50.0% of dMMR cases). While all cases with isolated PMS2 loss demonstrated intact nuclear staining in internal positive controls, suggesting against widespread technical failure, subtle technical issues (e.g., variable antibody sensitivity, antigen retrieval efficiency, or tissue fixation differences) cannot be entirely excluded without confirmatory testing. Therefore, we refrain from drawing mechanistic conclusions regarding the cause of isolated PMS2 loss. Repeat IHC with different antibody clones or confirmatory MSI analysis would be necessary to definitively distinguish true biological deficiency from potential technical artifact.

The combined loss of MLH1/PMS2 is typically attributed to somatic MLH1 promoter hypermethylation in sporadic CRCs, whereas the loss of MSH2/MSH6 more frequently suggests an underlying germline mutation (Lynch syndrome) (16). The absence of MSH2 loss in our cohort is notable but may be due to the relatively small sample size. Isolated loss of MSH6, observed in 16.7% of dMMR cases, similarly requires molecular confirmation to determine its etiology.

Our analysis revealed that dMMR CRCs possess a distinct clinicopathological profile compared to pMMR CRCs. We found that dMMR status was significantly and independently associated with right colon location, poor differentiation, and mucinous histology. The predilection for the proximal colon is a hallmark of dMMR CRCs, likely related to the different embryological origin and microenvironment of the right colon, which may be more susceptible to the CpG island methylator phenotype (CIMP) pathway that often leads to MLH1 silencing. The association with poor differentiation and mucinous histology reflects the high mutational burden in dMMR CRCs, which can lead to the accumulation of mutations in genes that govern differentiation and produce aberrant, often mucinous, tumor phenotypes.

A key finding of our research was the significant negative correlation between dMMR status and lymph node metastasis, suggesting a more favorable prognostic feature. Despite being poorly differentiated, dMMR CRCs appear to have a lower propensity for lymphatic spread. This observation has been reported in the literature, though our study did not assess survival outcomes (17). The robust host immune response triggered by the high number of neoantigens in these hypermutated tumors may contribute to their better prognosis, although tumor-infiltrating lymphocytes were not systematically quantified in this study. Although we observed a trend toward earlier TNM stage in the dMMR group, it did not reach statistical significance, possibly because of the limited number of dMMR cases in our study.

Multivariable analysis confirmed that right colon location, poor differentiation, and mucinous histology were independent predictors of dMMR status. Interestingly, while larger tumor size (≥ 5 cm) was associated with dMMR in univariate and correlation analyses, it did not remain an independent indicator in the multivariable model. This suggests that tumor size may be a confounding variable, closely related to other stronger predictors like location and histological type. The mucinous nature of many dMMR CRCs may contribute to their larger size without necessarily implying a more aggressive biological behavior.

When we analyzed the specific patterns of protein loss, we observed distinct associations. MLH1 loss (always with PMS2) was strongly tied to the classic sporadic dMMR phenotype: right-sided, poorly differentiated, and mucinous. Isolated PMS2 loss, which was the most frequent finding, shared many of these features, particularly right-sided location and poor differentiation, suggesting that these tumors, whether germline or somatic in origin, follow a similar biological pathway. In contrast, MSH6 loss, though only seen in 3 patients, showed a suggestive association with younger age and poor differentiation. This finding, while preliminary due to the very small sample size, aligns with the characteristics of Lynch syndrome, where MSH6 mutations are known to present at a later age than other mutations but still confer increased cancer risk. This underscores that not all dMMR CRCs are biologically identical; the underlying mechanism of deficiency (somatic vs. germline) can influence clinical presentation.

### Clinical implications

Our findings reinforce the critical role of universal IHC testing for MMR status in all newly diagnosed CRC patients, as recommended by current international guidelines (NCCN, ESMO, CAP). The distinct clinicopathological features associated with dMMR—particularly right-sided location, poor differentiation, and mucinous histology—can serve as valuable flags to raise suspicion for dMMR and prompt consideration of MMR testing, especially in resource-limited settings where universal testing may not be immediately feasible. However, these features should not replace universal testing; ideally, every CRC patient should undergo MMR status determination regardless of morphological characteristics. Nevertheless, because our study relied solely on IHC without confirmatory molecular analyses (such as MSI testing, BRAF V600E mutation, MLH1 promoter methylation, or germline sequencing), we cannot definitively distinguish between sporadic dMMR cases (usually due to MLH1 hypermethylation) and those with Lynch syndrome. This distinction has important implications for genetic counseling and family screening. Identifying specific loss patterns, such as MSH6 loss in younger patients, can suggest Lynch syndrome and guide timely referrals for genetic counseling, but definitive diagnosis requires germline sequencing. Most importantly, confirming dMMR status by IHC is a well-accepted method for identifying patients who are likely to benefit from immunotherapy; however, confirmation by MSI analysis may be considered in selected cases according to institutional guidelines.

## Limitations

This research has several limitations. First, it is a single-center study with a modest sample size, particularly the dMMR subgroup (*n* = 18). Consequently, the multivariable logistic regression analysis, although simplified using stepwise selection, may still be subject to overfitting (EPV = 6:1); the results should be considered exploratory and need validation in larger, independent cohorts. The limited sample size also restricts statistical power to detect more subtle associations and makes the analysis of rare loss patterns (e.g., MSH6) exploratory. Second, the cross-sectional design prevents us from drawing conclusions about patient survival or response to therapy; we could only assess correlations with pathological features at the time of diagnosis. Third, the borderline *P* values for some associations (e.g., lymph node metastasis, *P* = 0.052) were not adjusted for multiple comparisons; thus, they should be interpreted cautiously as hypothesis-generating rather than definitive. Fourth, and most importantly, our study did not include confirmatory molecular testing. Specifically, we did not perform MSI analysis (PCR or NGS) to confirm microsatellite instability status, nor did we conduct BRAF V600E mutation testing, MLH1 promoter methylation analysis, or germline sequencing for Lynch syndrome-associated genes. Consequently, we cannot determine which dMMR cases were sporadic (likely due to MLH1 hypermethylation) versus those with Lynch syndrome (germline mutations). Furthermore, the high frequency of isolated PMS2 loss observed in our study cannot be definitively attributed to any specific mechanism (sporadic vs. Lynch syndrome vs. technical artifact) without confirmatory molecular testing. Therefore, we refrain from drawing mechanistic conclusions regarding the cause of isolated PMS2 loss. Moreover, because we did not perform MSI PCR or NGS testing, we cannot exclude the possibility of rare discordant cases (estimated 1–5% of CRC) where IHC results may not align with functional microsatellite instability status. This may have minor implications for the precise classification of dMMR/pMMR in a small number of cases. Additionally, while internal positive controls were intact in all cases with isolated PMS2 loss, subtle technical factors (e.g., variable antibody sensitivity, antigen retrieval efficiency, or tissue fixation differences) cannot be entirely ruled out without repeat testing using alternative antibody clones or confirmatory MSI analysis. This distinction is critical for genetic counseling, family screening, and appropriate referral for immunotherapy. Therefore, our conclusions regarding Lynch syndrome screening and immunotherapy candidacy are based solely on IHC findings and should be interpreted with this limitation in mind. Furthermore, our findings should not be interpreted as advocating for selective MMR testing based solely on morphology; universal testing remains the standard of care, and the clinicopathological associations described here are intended only to raise awareness, not to replace guideline-recommended universal screening. Finally, the assessment of tumor-infiltrating lymphocytes (TILs), a key feature of dMMR CRCs, was not systematically quantified in this study.

## Conclusion

In conclusion, this study demonstrates a dMMR prevalence of 22.5% in our cohort of Chinese CRC patients, with isolated loss of PMS2 being the most common pattern. We confirm that dMMR CRCs exhibit a distinct phenotype characterized by right-sided location, poor differentiation, mucinous histology, larger tumor size, as well as a lower likelihood of lymph node metastasis. Right colon location, poor differentiation, and mucinous histology were identified as independent predictors of dMMR status. While our findings highlight the importance of routine IHC-based MMR testing for clinical and pathological characterization of dMMR CRC in the Chinese population, the lack of confirmatory molecular testing (MSI analysis, BRAF V600E, MLH1 methylation, or germline sequencing) limits our ability to distinguish sporadic from hereditary cases. Future studies with larger, multi-center cohorts as well as integrated genetic analysis are warranted to further elucidate the prognostic and predictive implications of specific MMR loss patterns and to validate the utility of IHC as a standalone test for Lynch syndrome screening and immunotherapy guidance.

## Data Availability

The original contributions presented in this study are included in the article/supplementary material, further inquiries can be directed to the corresponding author.
